# Immunoglobulin G subtypes-1 and 2 differentiate immunoglobulin G4-associated sclerosing cholangitis from primary sclerosing cholangitis

**DOI:** 10.1177/2050640620916027

**Published:** 2020-04-29

**Authors:** Miroslav Vujasinovic, Pia Maier, Hartwig Maetzel, Roberto Valente, Raffaella Pozzi-Mucelli, Carlos F Moro, Stephan L Haas, Karouk Said, Caroline S Verbeke, Patrick Maisonneuve, J-Matthias Löhr

**Affiliations:** 1 Department for Digestive Diseases, Karolinska University Hospital, Stockholm, Sweden; 2 Department of Abdominal Radiology, Karolinska University Hospital, Stockholm, Sweden; 3 Department of Pathology, Karolinska University Hospital, Stockholm, Sweden; 4 Department of Clinical Science, Intervention, and Technology (CLINTEC), Karolinska Institute, Stockholm, Sweden; 5 Department of Pathology, University of Oslo, Oslo, Norway; 6 Division of Epidemiology and Biostatistics, European Institute of Oncology IRCCS, Milan, Italy

**Keywords:** Autoimmune pancreatitis, immunoglobulin G4-associated cholangiatis, primary sclerosing cholangiatis, immunoglobulin G1, immunoglobulin G2

## Abstract

**Background:**

Autoimmune pancreatitis is a special form of chronic pancreatitis with strong lymphocytic infiltration and two histopathological distinct subtypes, a lymphoplasmacytic sclerosing pancreatitis and idiopathic duct centric pancreatitis. Immunoglobulin G4-associated cholangitis may be present at the time of autoimmune pancreatitis type 1 diagnosis or occur later over the course of the disease. Immunoglobulin G4 is considered reliable but not an ideal marker for diagnosis of autoimmune pancreatitis type 1 with reported sensitivity between 71–81%. It is essential to differentiate sclerosing cholangitis with autoimmune pancreatitis from primary sclerosing cholangitis as the treatment and prognosis of the two diseases are totally different. It was the aim of the study to find a marker for immunoglobulin G4-associated cholangitis that would distinguish it from primary sclerosing cholangitis.

**Patients and methods:**

We performed a retrospective analysis of patients with autoimmune pancreatitis at our outpatient clinic. Patients from the primary sclerosing cholangitis registry were taken as a control group. Blood samples for the measurement of immunoglobulin subclasses were analysed at the time of diagnosis.

**Results:**

Patients with autoimmune pancreatitis and immunoglobulin G4-associated cholangitis had higher values of immunoglobulin G2 when compared to autoimmune pancreatitis alone or primary sclerosing cholangitis with a high specificity (97%) and high positive predictive value (91%). In patients with normal or low immunoglobulin G2 or immunoglobulin G4, a high level of immunoglobulin G1 indicated primary sclerosing cholangitis.

**Conclusion:**

Immunoglobulin G1 and immunoglobulin G2 can distinguish patients with immunoglobulin G4-associated cholangitis from those with primary sclerosing cholangitis.

## Key summary

- Autoimmune pancreatitis (AIP) is a special form of chronic pancreatitis with a strong lymphocytic infiltration and two histopathological distinct subtypes, lymphoplasmacytic sclerosing pancreatitis (type 1) and idiopathic duct centric pancreatitis (type 2).
- IgG4-associated cholangiopathy (IAC) may be present at the time of AIP type 1 diagnosis or occur later over the course of the disease.
- It is essential to differentiate sclerosing cholangitis with AIP from primary sclerosing cholangitis (PSC) as the treatment and prognosis of the two diseases are totally different.
- IgG4 is considered reliable but not an ideal marker for diagnosis of AIP type 1 with reported sensitivity between 71–81%. IgG1 and IgG2 can distinguish patients with AIP-related cholangitis (IAC) from those with PSC.

## Introduction

Autoimmune pancreatitis (AIP) is a form of chronic pancreatitis with strong lymphocytic infiltration and fibrosis as the pathological characteristics and two histopathological subtypes: lymphoplasmacytic sclerosing pancreatitis (AIP type 1) and idiopathic duct centric pancreatitis (AIP type 2).^1^ It was first described by Sarles et al. in 1961^2^ and coined as AIP in 1995 when clinicopathological similarities to autoimmune hepatitis were described.^3^ It soon became apparent that AIP type 1 is part of a systemic disease defined by fibrosclerosis and elevated immunoglobulin G4 (IgG4) known as IgG4-related disease (IgG4-RD).^4^ These systemic manifestations, from the AIP type 1 perspective, are called ‘other organ involvement’ (OOI) and of these, a special form of cholangitis, then called immune or IgG4-associated cholangitis (IAC) is the most frequent manifestation.^5^

Primary sclerosing cholangitis (PSC) is a chronic cholestatic liver disease characterised by progressive destruction of the bile ducts and development of cirrhosis.^6^ Most of the small, uncontrolled trials showed no significant benefit with the use of oral steroids in patients with PSC.^6^ On the other hand, AIP is a disease with a good response to steroid treatment.^5^ Diagnosing IAC and differentiating it from PSC is a major clinical challenge.^7^ IAC may precede AIP, be present at the time of AIP diagnosis or it can occur later.^8^ Similar to the endoscopic retrograde cholangiopancreatography/ magnetic resonance cholangiopancreatography (ERCP/MRCP) classification of PSC,^9^ a classification for cholangitis in AIP was proposed^10^ that became part of the Japanese guidelines.^11^ Nevertheless, to establish the diagnosis by imaging alone is difficult when signs of AIP are sparse or absent.

The diagnosis of AIP and accompanying OOI can be made according to the international consensus diagnostic criteria (ICDC)/Honolulu criteria^12^ or a modification^13^ of the original M-ANNHEIM criteria.^14^ IgG4 is useful but not an ideal biomarker for diagnosis of AIP type 1.^15^ Several other serum markers for AIP have been determined, including autoantibodies against lactoferrin, carbonic anhydrase II, the serine protease inhibitor kazal type 1 gene (SPINK1), ubiquitin, trypsinogens and N-glykan.^16–18^ However, none of these are globally available for routine clinical testing. Furthermore, there are no markers available to identify IAC as part of the AIP type 1/IgG4-RD syndrome either. From a clinical perspective, it is essential to differentiate cholangitis with AIP from PSC, as the treatment and prognosis of these two diseases are different.^19^

We tested all IgG subclasses in order to determine their usefulness to differentiate AIP with IAC from PSC. Here we describe elevated levels of IgG2 as a marker for IAC (that are not present in PSC) and elevated levels of IgG1 in patients with PSC (significantly higher compared to AIP patients).

## Patients and methods

We performed a retrospective analysis of patients with AIP at the outpatient clinic at the Department of Digestive Diseases of Karolinska University Hospital in Stockholm, Sweden between September 2007–October 2018. Patients from the PSC registry from the department with proven histology were taken as a control group. The demographic, immunological and clinical characteristics of both groups were recorded and analysed (Table 1). The diagnosis of AIP was made according to the ICDC.^12^ Remission was defined as an absence of clinical symptoms and resolution of the pancreatic and extrapancreatic manifestations on imaging. Relapse was defined as a recurrence of symptoms with the reappearance of pancreatic or extrapancreatic abnormalities on imaging. The following IgG subclasses serum levels were considered as normal: total IgG: 6.7–14.5 g/l; IgG1: 2.8–8.0 g/l; IgG2: 1.15–5.7 g/l; IgG3: 0.24–1.25 g/l; IgG4: 0.05–1.25 g/l.

**Table 1.** Characteristics of patients with primary sclerosing cholangitis (PSC), autoimmune pancreatitis (AIP) and immunoglobulin G4 (IgG4)-associated cholangitis (IAC).

	PSC *n* (%)	AIP *n* (%)	*p*-Value^a^	AIP without IAC *n* (%)	AIP with IAC *n* (%)	*p*-Value^a^
All	73 (100)	69 (100)		14 (100)	55 (100)	
Sex						
Males	51 (69.9)	38 (55.1)		4 (28.6)	34 (61.8)	
Females	22 (30.1)	31 (44.9)	0.08	10 (71.4)	21 (38.2)	0.04
Age						
<40	33 (45.2)	17 (24.6)		4 (28.6)	13 (23.6)	
40–49	16 (21.9)	12 (17.4)		5 (35.7)	7 (12.7)	
50–59	7 ( 9.6)	7 (10.1)		1 (7.1)	6 (10.9)	
60–69	16 (21.9)	19 (27.5)		2 (14.3)	17 (30.9)	
70+	1 (1.4)	14 (20.3)	0.001	2 (14.3)	12 (21.8)	0.33
IgG2						
Mean ± SD (g/l)	3.3 ± 1.2	5.1 ± 2.4		4.6 ± 2.0	5.2 ± 2.4	
Median (range) (g/l)	3.3 (0.8–6.1)	4.5 (1.7–13.1)	<0.0001	4.6 (1.7–8.6)	4.5 (1.9–13.1)	0.57
Low (<1.15 g/l)	1	0		0	0	
Normal (1.15–5.7 g/l)	70	46		11	35	
High (>5.7 g/l)	2	21	<0.0001	3	18	0.52
Sensitivity	21/67	31% (21–44)		18/53	34% (22–48)	
Specificity	71/73	97% (90–100)		11/14	79% (49–95)	
Positive predictive value	21/23	91% (72–99)		18/21	86% (64–97)	
Negative predictive value	71/117	61% (51–70)		11/46	24% (14–38)	
IgG4						
Mean ± SD (g/l)	0.4 ± 0.4	2.0 ± 3.9		1.2 ± 1.2	2.2 ± 4.3	
Median (range) (g/l)	0.4 (0.0–1.6)	0.9 (0.1–26.2)	<0.0001	0.8 (0.2–4.5)	0.9 (0.1–26.2)	0.76
Low (<0.05 g/l)	8 (11.0)	0 (0.0)		0 (0.0)	0 (0.0)	
Normal (0.05–1.25 g/l)	62 (84.9)	39 (56.5)		9 (64.3)	30 (54.5)	
High (>1.25 g/l)	3 (4.1)	30 (43.5)	<0.0001	5 (35.7)	25 (45.5)	0.56
IgG2 and IgG4						
Low or normal IgG2 and IgG4	68	30		8	22	
High IgG2 or high IgG4	5	39^b^	<0.0001	6	33	0.37
Sensitivity	39/69	57% (44–68)		33/55	60% (46–73)	
Specificity	68/73	93% (85–98)		8/14	57% (29–82)	
Positive predictive value	39/44	89% (75–96)		33/39	85% (69–94)	
Negative predictive value	68/98	69% (59–78)		8/30	27% (12–46)	

Ig: immunoglobulin; SD: standard deviation.

95% Confidence intervals for the sensitivity, specificity, positive predictive value and negative predictive value calculated using the Binomial (Clopper-Pearson) exact method. No differences in the distribution of IgG, IgG1 and IgG3 between groups were found (see Supplementary Material Table 1).

^a^*p*-Value calculated with the non-parametric Wilcoxon test for continuous variables and the Fisher exact test for categorical variables.

^b^18 patients had normal IgG2 and High IgG4, 9 patients had High IgG2 and normal IgG4, 12 patients had High IgG2 and High IgG4.

### Ethics

The study was approved by the local ethics committee (EPN Regionala etikprövningsnämnden Stockholm Dnr. 2014/902-31/2; 2016/1571-31, 5 December 2016).

### Statistics

Differences in the distribution of patient characteristics across groups were assessed with the Fisher exact test for categorical variables and the non-parametric Wilcoxon rank-sum test (Mann-Whitney U test) for continuous variables. The prognostic ability of IgGs to distinguish AIP from PSC was assessed using receiver-operating characteristic (ROC) curves, generated by plotting the sensitivity vs 1-specificity, giving the ideal test both a sensitivity and a specificity equal to one. The area under the curve (AUC) was used as a measure of the diagnostic efficiency of the test. The sensitivity, specificity, positive predictive value (PPV) and negative predictive value (NPV) of single and combined IgGs were calculated with respective 95% confidence intervals (CIs) based on the binomial (Clopper-Pearson) exact method. The diagnostic performance of IgG2, IgG4 and their combination to differentiate AIP from PSC was further assessed using a logistic regression model, in which IgG2 and IgG4 were dichotomised as high (>5.7 g/l for IgG2 and >1.25 g/l for IgG4) vs low or normal. Changes in the likelihood ratio value (LRΔχ^2^) from models including and excluding the variable of interest were used to quantitatively measure the diagnostic performance of IgG4 alone, IgG2 alone, and of the addition of IgG2 or IgG4 to the other biomarker.

The analyses were performed with SAS software version 9.4 (SAS Institute, Cary, North Carolina, USA). All *p*-values were two-sided. A *p*-value less than 0.05 was considered statistically significant.

## Results

From the patient registries, we included 142 patients where all IgG subclasses were measured, 69 with AIP type 1 and 73 with PSC (Table 1 and Supplementary Material Table s1).

### AIP vs PSC

**Figure 1.**
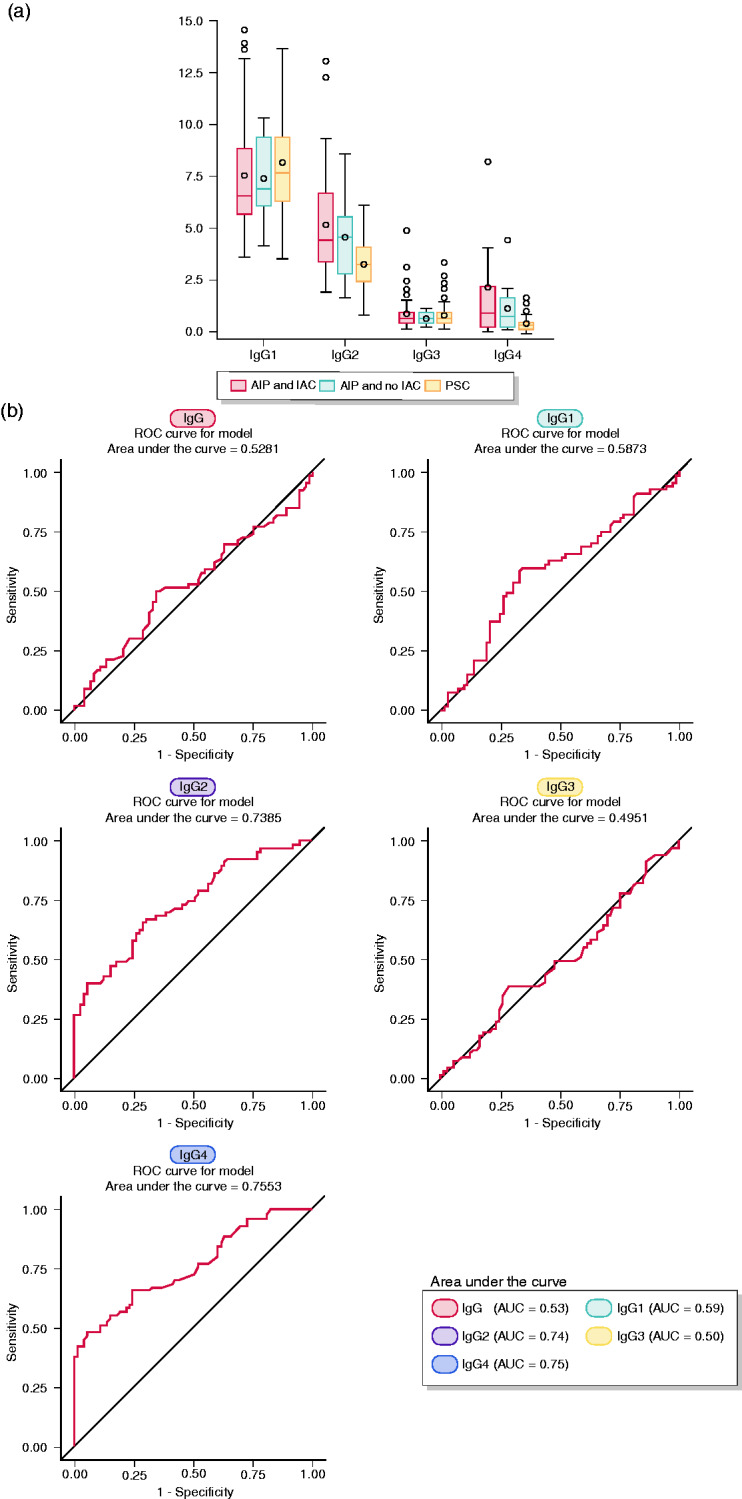
(a) Distribution of total immunoglobulin (Ig)G and single IgG subclasses in patients with autoimmune pancreatitis (AIP) and no IgG4-associated cholangitis (IAC), in patients with AIP and IAC, and in patients with primary sclerosing cholangitis (PSC) is shown using box and whisker plots. The box spans the interquartile range (25% and 75% percentiles). A circle and a vertical line inside the box mark the mean and median respectively. The whiskers are the two lines outside the box that extend to the highest and lowest observations. If any, outliers’ values (±3 standard deviations) are represented by circles above or below the whiskers. (b) Diagnostic performance of total IgG and of the various IgG subclasses for the distinction between AIP and PSC was assessed using receiver-operating characteristic (ROC) curve analysis, and accuracy was measured by the area under the curve (AUC). Accuracy is considered ‘excellent’ when AUC is comprised between 0.90–1.0,’ good’ when AUC is comprised between 0.8–0.9, ‘fair’ when AUC is comprised between 0.7–0.8, ‘poor’ when AUC is comprised between 0.6–0.7.

Firstly, we compared the distribution of characteristics of patients with PSC with that of patients with AIP (Table 1, left side). Of all IgG subclasses, only IgG2 (*p*<0.0001) and IgG4 (*p*<0.0001) distinguish patients with AIP from patients with PSC. Using ROC curve analysis, the AUC for the differentiation between AIP and PSC was 0.74 for IgG2 and 0.75 for IgG4, and was lower than 0.6 for total IgG, IgG1 and IgG3 (Figure 1(a)). The diagnostic performance of IgG2 and of the combination of IgG2 and IgG4 for the distinction of patients with PSC and AIP was further assessed by means of sensitivity, specificity, PPV and NPV. High IgG2 has a high specificity (97%) and PPV (91%) to identify patients with AIP, but a low sensitivity (31%). The combination of high IgG2 and IgG4 retains similar specificity (93%) and PPV (89%) but increases the sensitivity to 57%. No difference in the distribution of IgG, IgG1 and IgG3 between groups was found (Supplementary Material Table s1). The diagnostic performance of IgG2 and IgG4 to differentiate AIP from PSC was further quantified by the likelihood ratio test: The LRΔχ^2^ with one degree of freedom (df) was 22.6 (*p*<0.0001) for IgG4 compared to 34.5 ((*p*<0.0001) for IgG4. Both addition of IgG4 to IgG2 (LRΔχ^2^ (1 df) = 25.3, *p*<0.0001) and IgG2 to IgG4 (LRΔχ2 (1 df) = 13.5, (*p* = 0.0002) increased significantly the diagnostic performance compared to IgG2 and IgG4 alone (Supplementary Material Table s3).

### AIP with AIC vs AIP without AIC

Similar analysis for the comparison of AIP patients with and without IAC was performed (Table 1, right side). Of all variables, only gender distribution was significantly different between the two groups: 34 (61.8%) of the 55 patients with IAC were males while 10 (71.4%) of the 14 patients with no IAC were females (*p* = 0.04).

The distribution of IgG levels in the three patients’ groups (AIP with AIC, AIP without AIC and PSC) is displayed in Figure 1(a), illustrating lower IgG2 and IgG4 levels in patients with PSC than in patients with AIP, with the diagnostic performance pictured in Figure 1(b).

### Distinction of PSC and AIP using IgG1, IgG2 and IgG4

Furthermore, we evaluated the diagnostic performance of IgG, IgG1 and IgG3 among patients with low or normal IgG2 and IgG4 (Table 2 and Supplementary Material Table s2). In this subgroup, IgG1 was significantly higher in patients with PSC (mean ± standard deviation (SD), 8.2 ± 2.6 g/l) than in patients with AIP (6.7 ± 2.2 g/l) (*p* = 0.006), while no difference was observed for IgG3. Furthermore, high IgG2 or IgG4 levels were observed in 31% of the patients and identified those with AIP (Supplementary Material Table s3: PPV = 89%, 39/44); high IgG1 with low or normal IgG2 and IgG4 levels was observed in 27.5% of the patients and identified those with PSC (Supplementary Material Table s3: PPV = 85%, 33/39 and Supplementary Material Table s4). The condition of the remaining 59 patients (41.5% of our series) with low or normal IgG1, IgG2 and IgG4 remained undetermined.

**Table 2.** Characteristics of the 98 patients with low or normal immunoglobulin (Ig)G2 and IgG4.

	PSC	AIP	*p*-Value^a^	AIP and no IAC	AIP and IAC	*p*-Value^a^
All	68 (100)	30 (100)		8 (100)	22 (100)	
Sex						
Males	47 (69.1)	11 (36.7)		2 (25.0)	9 (40.9)	
Females	21 (30.9)	19 (63.3)	**0.004**	6 (75.0)	13 (59.1)	0.67
Age						
<40	32 (47.1)	13 (43.3)		2 (25.0)	11 (50.0)	
40–49	14 (20.6)	5 (16.7)		2 (25.0)	3 (13.6)	
50–59	6 ( 8.8)	3 (20.0)		1 (12.5)	2 (9.1)	
60–69	15 (22.1)	5 (16.7)		2 (25.0)	3 (13.6)	
70+	1 (1.5)	4 (13.3)	0.22	1 (12.5)	3 (13.6)	0.72
IgG						
Mean ± SD (g/l)	13.6 ± 3.4	11.7 ± 3.6		10.6 ± 4.5	12.1 ± 3.2	
Median (range)	13.2 (7.0–25.8)	11.4 (0.3–20.3)	**0.007**	11.8 (0.3–16.0)	11.0 (8.6–20.3)	1.00
Low (<6.1 g/l)	0 (0.0)	1 (3.3)		1 (12.5)	0 (0.0)	
Normal (6.1–14.5 g/l)	45 (66.2)	24 (80.0)		6 (75.0)	18 (81.8)	
High (>14.5 g/l)	23 (33.8)	5 (16.7)	0.08	1 (12.5)	4 (18.2)	0.35
IgG1						
Mean ± SD (g/l)	8.2 ± 2.6	6.7 ± 2.2		6.9 ± 1.7	6.7 ± 2.4	
Median (range)	7.8 (3.5–17.0)	6.3 (3.7–13.6)	**0.006**	6.4 (4.2–9.6)	6.2 (3.7–13.6)	0.53
Low (<2.8 g/l)	0 (0.0)	0 (0.0)		0 (0.0)	0 (0.0)	
Normal (2.8–8.0 g/l)	35 (51.5)	24 (80.0)		6 (75.0)	18 (81.2)	
High (>8.0 g/l)	33 (48.5)	6 (20.0)	**0.01**	2 (25.0)	4 (18.2)	0.65
IgG3						
Mean ± SD (g/l)	0.8 ± 0.5	0.8 ± 0.8		0.7 ± 0.3	0.8 ± 1.0	
Median (range)	0.7 (0.2–3.4)	0.6 (0.2–4.9)	0.52	0.6 (0.3–1.2)	0.6 (0.2–4.9)	0.61
Low (<0.24 g/l)	3 (4.4)	1 (3.3)		0 (0.0)	1 (4.5)	
Normal (0.24–1.25 g/l)	56 (82.4)	27 (90.0)		8 (100)	19 (86.4)	
High (>1.25 g/l)	9 (13.2)	2 ( 6.7)	0.79	0 (0.0)	2 (9.1)	1.00

AIP: autoimmune pancreatitis; IAC: immune-associated cholangitis; PSC: primary sclerosing cholangitis; SD: standard deviation.

^a^*p*-Values calculated with the non-parametric test of median for continuous variables and the Fisher exact test for categorical variables.*p*-Value less than 0.05 was considered statistically significant and are in bold.

### Association between presence of IAC and IgG levels and disease features

A higher proportion of AIP patients with IAC than AIP patients with no IAC required stenting (56.4% vs 7.1%, (*p* = 0.0009) or had a relapse (47.3% vs 7.1%, (*p* = 0.006). Relapse was also more frequent in AIP patients with a high IgG4 level than in patients with a normal IgG4 level (53.3% vs 28.2%, (*p* = 0.047). Data on the association between the presence of IAC and IgG levels and disease remission and treatment in patients with AIP are presented in Supplementary Material Table s2.

### Association between IgG subclasses and response to therapy

In only three patients, we noticed a very high IgG4 (>5 g/l, Supplementary Material Table s2). The first patient had IgG4 of 26.2 and IgG2 of 12.2. A second patient (treated with corticosteroids (CST), CellCept and cyclosporine) had IgG4 of 8.2. In a third patient, the IgG4 was 19.4 g/l. We had no IgG2 in the diagnosis. In controls after CST IgG2 was 11.2 and IgG4 0.27, respectively. In a total of 10 patients we had follow-up values after therapy. In six patients, elevated IgG2 normalised after the treatment, and in four patients IgG2 remained elevated despite CST treatment.

**Figure 2.**
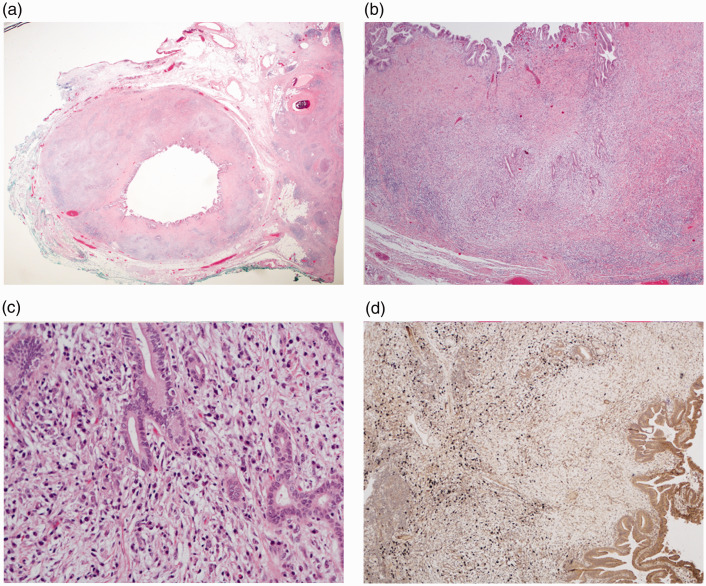
(a) Immunoglobulin (Ig)G4-associated cholangitis (IAC) with concentric wall thickening of the extrapancreatic common bile duct. Note the rim of adjacent pancreas (right) showing atrophy, fibrosis and peripancreatitis as a consequence of autoimmune pancreatitis (AIP) type 1 (hemotoxylin and eosin (H&E) 5x). (b) Marked thickening of the bile duct wall with transmural inflammatory cell infiltration (H&E, 10x). (c) High-power magnification reveals dense inflammatory cell infiltration with dominance of plasma cells and relatively modest fibrosis, findings that are in contrast with those typically found in primary sclerosing cholangitis (PSC) (H&E, 40x). (d) Immunohistochemical staining for IgG4 (brown) reveals prominent infiltration with IgG4+ plasma cells, also in deeper layers of the bile duct wall (10x).

**Figure 3.**
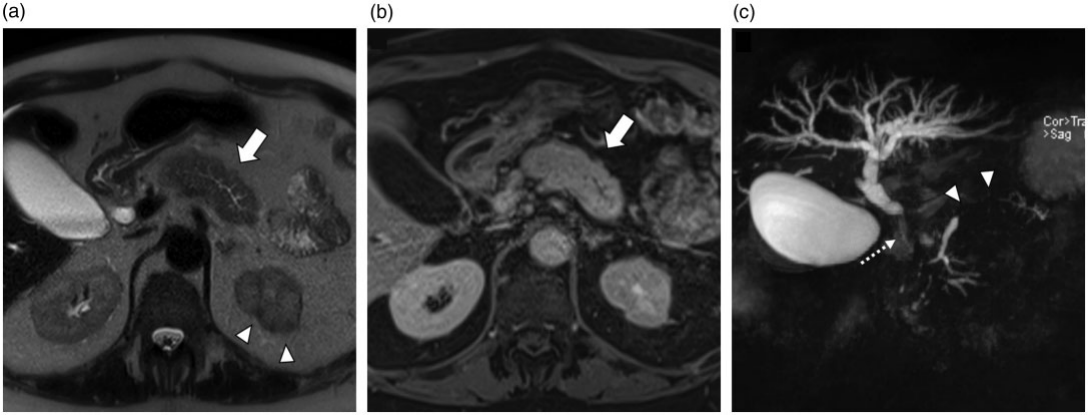
Magnetic resonance imaging (MRI) of a patient with autoimmune pancreatitis (AIP) type 1 and other organ involvement (OOI). The T2-weighted axial image (a) and the venous phase (b) show a diffuse enlargement of the pancreas (also known as ’sausage pancreas’) (arrows). The maximum intensity projection (MIP) reformatted image of the magnetic resonance cholangiopancreatography (MRCP) sequence shows a long stricture of the main pancreatic duct (MPD) without upstream dilatation (arrowheads), and a stricture of the distal common bile duct (CBD) with upstream dilatation (dotted thin arrow). Furthermore, there are hypointense cortical lesions in the left kidney (a) (arrowheads) and worse contrast-enhancement in the renal parenchyma in the venous phase (b).

**Figure 4.**
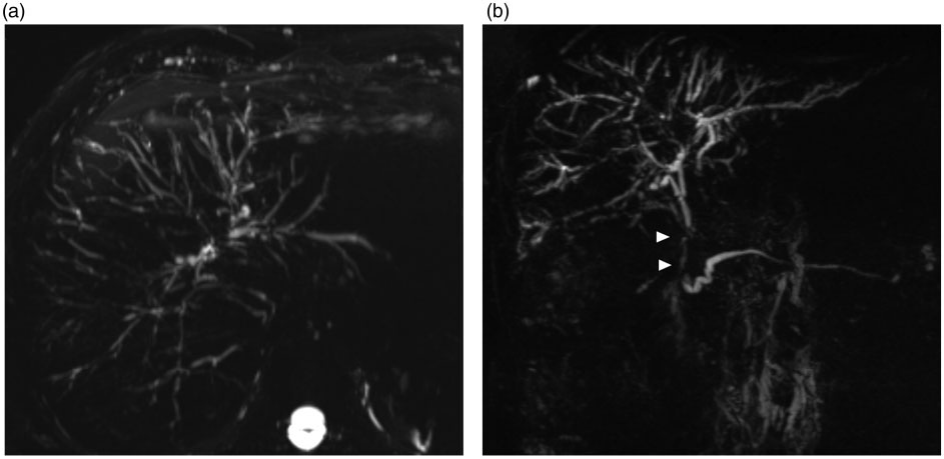
Patient with primary sclerosing cholangitis (PSC). The maximum intensity projection (MIP) reformatted images of the MRCP-sequences in the axial (a) and coronal plane (b) show multiple and short biliary strictures in several liver segments. The coronal image shows also a stricture of the distal common bile duct (CBD) (arrowheads).

Histology revealed the typical features of IAC (Figure 2(a)–(c)) including positive immunohistochemistry for IgG4 in the common bile duct (Figure 2(d)). In all patients, the typical images of AIP could be obtained by magnetic resonance imaging and of IAC by MRCP (Figure 3); whilst PSC gave a different albeit typical imaging pattern (Figure 4).

## Discussion

AIP type 1 is part of the systemic IgG4-RD with IAC as the most frequent OOI.^5^ Here we describe elevated levels of IgG2 as markers of IAC that were not present in PSC or AIP without cholangitis.

For AIP type 1, the only clinically available serum marker is elevated total IgG or IgG4 in the blood, despite efforts to identify other more specific markers. Slight elevations of IgG4 concentration are seen also in other diseases such as pancreatic cancer.^20^ Furthermore, between 3–30% of IgG4-RD patients have normal serum IgG4 concentrations;^21^ however, they may suffer from significant disease including AIP with IAC.^5^ Different IgGs bind to different receptors and distinct genes encode six human receptors for IgG.^22^ IgG1 and IgG3 bind to all human receptors for IgG; IgG2 binds not only to FcγRIIA_H131_, but also has a lower affinity to FcγRIIA_R131_ and FcγRIIIA_V158_; IgG4 binds to FcγRI, FcγRIIA, IIB and IIC and FcγRIIIA_V158_.^22^ Consequently, IgG1 and IgG2, distinguishing IAC, bind to different receptors than IgG4.

In other autoimmune diseases such as systemic lupus erythematosis (SLE), differential expression/elevation of IgG subclasses has been reported with significantly higher levels of IgG1, IgG2 and IgG3 in SLE as compared to healthy controls,^23^ however, with no correlation to distinct clinical features. Elevated serum IgG subclass levels were also reported in a series encompassing several autoimmune diseases, including primary Sjögren syndrome (SS), systemic sclerosis, SLE and primary biliary cirrhosis (PBC) and showed a significantly increased level of IgG1 and IgG3 compared with those in healthy controls.^24^ In this study, IgG2 was significantly reduced in SS and SLE compared to healthy subjects and indifferent in PBC.^24^ In another study in autoimmune rheumatological disease,^25^ elevated levels of IgG1 were present in 55% of primary SS, 50% of secondary SS and 30% in the lupus and myositis groups, respectively (Supplementary Material Table s5).

Making the distinction between isolated SS (Mikulicz’s disease) and IgG4-RD, IgG2 was only found elevated in those having IgG4-RD, including AIP, but not in those with isolated SS.^26^

Similarly, significant elevations of serum IgG2 and tissue IgG2 plasma cells were described in orbital IgG4-RD in comparison with non-IgG4 orbital inflammation, suggesting that IgG2 may play a role in IgG4-RD.^27^ Elevation of serum IgG2 was described in two cases as a precursor to classical IgG4-RD in a patient with periorbital xanthogranulomatosis^28^ and in another patient with tubulointerstitial nephritis.^29^ There are other rare conditions described resembling IgG4-RD but they are negative for IgG4, both in serum and tissue, while IgG2 was again significantly elevated in tubulointerstitial nephritis.^30,31^ Patients with these conditions and elevated IgG2 are clearly clinically distinguishable from patients with IAC with and without AIP or PSC.

Retrospective analysis and lack of data on serum values of IgG subclasses before and after steroid treatment are the main limitations of the study. It is worth noticing that IgG subclasses can be slightly elevated in different groups of patients, but the differentiation of pancreatic cancer and classic chronic pancreatitis can be determined clinically.

From a clinical perspective, to positively identify a chronic cholangitis as being part of the IgG4-RD syndrome, i.e. IAC vs PSC is of the utmost significance. PSC does not respond to steroids or even biologicals^32^ in contrast to IAC^11^ which, in selected cases, can be treated by steroids only without stenting.^33^ Imaging findings of AIP-IAC and PSC on endoscopic retrograde cholangiography (ERC), magnetic resonance cholangiography (MRC) and computed tomography (CT) showed that the combination of ERC and MRC with cross-sectional images, may be helpful in differentiating between AIP, IAC and PSC.^19^ High serum IgG2 in those who are IgG/IgG4 positive and elevated IgG1 in those who have low or normal IgG2 and IgG4, indicating PSC, in this context can be considered an additional aid in establishing the one condition and/or excluding the other.

## Conclusion

High IgG2 or IgG4 levels identify patients with AIP, while high IgG1 in those with low or normal IgG2 and IgG4 levels identifies patients with PSC.

## Supplemental Material

sj-pdf-1-ueg-10.1177_2050640620916027 - Supplemental material for Immunoglobulin G subtypes-1 and 2 differentiate immunoglobulin G4-associated sclerosing cholangitis from primary sclerosing cholangitis Click here for additional data file. Supplemental material, sj-pdf-1-ueg-10.1177_2050640620916027 for Immunoglobulin G subtypes-1 and 2 differentiate immunoglobulin G4-associated sclerosing cholangitis from primary sclerosing cholangitis by Miroslav Vujasinovic, Pia Maier, Hartwig Maetzel, Roberto Valente, Raffaella Pozzi-Mucelli, Carlos F Moro, Stephan L Haas, Karouk Said, Caroline S Verbeke, Patrick Maisonneuve and J-Matthias Löhr in United European Gastroenterology Journal
