# Supplementary material for: Immunoglobulin G subtypes-1 and 2 differentiate immunoglobulin G4-associated sclerosing cholangitis from primary sclerosing cholangitis
Source: United European Gastroenterol J. 2020 Apr 29;8(5):584–93. doi: 10.1177/2050640620916027 (PMC7268946; doi:10.1177/2050640620916027)
Supplement: sj-pdf-1-ueg-10.1177_2050640620916027 - Supplemental material for Immunoglobulin G subtypes-1 and 2 differentiate immunoglobulin G4-associated sclerosing cholangitis from primary sclerosing cholangitis [file sj-pdf-1-ueg-10.1177_2050640620916027.pdf]

Supplementary Tables

Table s1. Characteristics of patients with PSC, AIP and IAC

	PSC N (%)	AIP N (%)	P-value*	AIP and no IAC N (%)	AIP with IAC N (%)	P-value*
**ALL**	73 (100)	69 (100)		14 (100)	55 (100)	
**IgG**						
Mean±SD (g/L)	13.8±3.4	13.7±4.3		12.8±4.5	14.0±4.3	
Median [range]	13.5 [7.0-25.8]	12.6 [0.3-26.9]	0.57	12.6 [0.3-19.7]	12.9 [8.4-26.9]	0.99
Low (<6.1 g/L)	0 (0.0)	1 (1.4)		1 (7.1)	0 (0.0)	
Normal (6.1-14.5 g/L)	45 (61.6)	41 (59.4)		8 (57.1)	33 (60.0)	
High (>14.5 g/L)	28 (38.4)	24 (34.8)	0.79	5 (35.7)	19 (34.5)	0.30
**IgG1**						
Mean±SD (g/L)	8.2±2.6	7.6±2.7		7.5±1.8	7.6±2.9	
Median [range]	7.7 [3.5-17.0]	6.7 [3.7-17.2]	0.07	6.9 [4.2-10.3]	6.6 [3.7-17.2]	0.53
Low (<2.8 g/L)	0 (0.0)	0 (0.0)		0 (0.0)	0 (0.0)	
Normal (2.8-8.0 g/L)	38 (52.1)	44 (63.8)		9 (64.3)	35 (63.6)	
High (>8.0 g/L)	35 (47.9)	23 (33.3)	0.12	5 (35.7)	18 (32.7)	1.00
**IgG3**						
Mean±SD (g/L)	0.8±0.6	0.9±0.7		0.7±0.3	0.9±0.8	
Median [range]	0.7 [0.2-3.4]	0.7 [0.2-4.9]	0.92	0.7 [0.3-1.2]	0.7 [0.2-4.9]	0.86
Low (<0.24 g/L)	3 (4.1)	3 (4.3)		0 (0.0)	3 (5.5)	
Normal (0.24-1.25 g/L)	60 (82.2)	56 (81.2)		14 (100)	42 (76.4)	
High (>1.25 g/L)	10 (13.7)	8 (11.6)	0.94	0 (0.0)	8 (14.5)	0.27

Primary Sclerosing Cholangitis (PSC); Autoimmune Pancreatitis (AIP); Immune-associated cholangitis (IAC); Standard deviation (SD)
*P-values calculated with the non-parametric Wilcoxon test for continuous variables and the Fisher exact test for categorical variables

Table s2. Association between presence of IAC and IgG levels and disease remission and treatment in patients with AIP

	AIP and no IAC N (%)	AIP with IAC N (%)	P-value*	IgG2 ≤5.7g/L N (%)	IgG2 >5.7g/L N (%)	P-value*	IgG4 ≤1.25g/L N (%)	IgG4 >1.25g/L N (%)	P-value*
**All**	**14 (100)**	**55 (100)**		**48 (100)**	**21 (100)**		**39 (100)**	**30 (100)**	
**Clinical remission****									
Without treatment	7 (50.0)	39 (70.9)		30 (62.5)	16 (76.2)		29 (74.4)	17 (46.7)	
On or after treatment	6 (42.9)	9 (16.4)		12 (25.0)	3 (14.3)		7 (17.9)	8 (26.7)	
No / dead	0 (0.0)	7 (12.7)	0.05	5 (10.4)	2 (9.5)	0.61	3 (7.7)	4 (13.3)	0.43
**Treatment**									
No	4 (28.6)	5 (9.1)		7 (14.6)	2 (9.5)		5 (12.8)	4 (13.3)	
Yes	10 (71.4)	50 (90.9)	0.08	41 (85.4)	19 (90.5)	0.71	34 (87.2)	26 (86.7)	1.00
**Steroids**									
No	5 (35.7)	14 (25.5)		12 (25.0)	7 (33.3)		13 (33.3)	6 (20.0)	
Yes	9 (64.3)	41 (74.5)	0.51	36 (75.0)	14 (66.7)	0.56	26 (66.7)	24 (80.0)	0.28
**Azathioprine**									
No	11 (78.6)	49 (89.1)		41 (85.4)	19 (90.5)		34 (87.2)	26 (86.7)	
Yes	3 (21.4)	6 (10.9)	0.37	7 (14.6)	2 (9.5)	0.71	5 (12.8)	4 (13.3)	1.00
**Stent**									
No	13 (92.9)	24 (43.6)		26 (54.2)	11 (52.4)		24 (61.5)	13 (43.3)	
Yes	1 (7.1)	31 (56.4)	**0.0009**	22 (45.8)	10 (47.6)	1.00	15 (38.5)	17 (56.7)	0.15
**Surgery**									
No	12 (85.7)	46 (83.6)		40 (83.3)	18 (85.7)		32 (82.1)	26 (86.7)	
Yes	2 (14.3)	9 (16.4)	1.00	8 (16.7)	3 (14.3)	1.00	7 (17.9)	4 (13.3)	0.75
**Relapse**									
No	13 (92.9)	29 (52.7)		30 (62.5)	12 (57.1)		28 (71.8)	14 (46.7)	
Yes	1 (7.1)	26 (47.3)	**0.006**	18 (37.5)	9 (42.9)	0.79	11 (28.2)	16 (53.3)	**0.047**

*P-values calculated with Fisher exact test; **data missing for 1 patient

Table s3. Prognostic information of IgG2, IgG4 and their combination

Model	Comparison High vs Low	Odds Ratio (95% CI)	LRΔχ2	DF	P-value
IgG2	>5.7 vs ≤5.7	15.5 (3.48-69.3)	22.7	1	<0.0001
IgG4	>1.25 vs ≤1.25	17.9 (5.14-62.6)	34.5	1	<0.0001
IgG2 +	>5.7 vs ≤5.7	11.4 (2.39-54.2)	48.0	2	<0.0001
IgG4	>1.25 vs ≤1.25	14.6 (4.06-52.6)			
IgG2+IgG4 vs IgG2			25.3	1	<0.0001
IgG2+IgG4 vs IgG4			13.5	1	0.0002

The prognostic contribution of high IgG2 (>5.7g/l) and high IgG4 (>1.25g/l) were evaluated by the change in Likelihood ratio values (LRΔχ2) assessed by logistic regression.

Table s4: Distinction of PSC and AIP using IgG1, IgG2 and IgG4

	Total N (%)	PSC N (%)	AIP N (%)	P-value
**All**	142 (100)	73 (100)	69 (100)	
**High IgG2 or IgG4**	44 (31.0)	5 (6.9)	39 (56.5)	
**Low/normal IgG1, IgG2, IgG4**	59 (41.5)	35 (47.9)	24 (34.8)	
**High IgG1, low/normal IgG2 and IgG4**	39 (27.5)	33 (45.2)	6 (8.7)	<0.0001

Table s5: Comparison of studies investigating IgG subclasses, especially IgG2, in syndromes with and without IgG4-RD, both in serum and in tissue

Author	Year	Pat n =	Disease/Syndrome	IgG	IgG1	IgG2	IgG3	IgG4	Ref
**Blanco**	1992	65	SS, SLE vs. HC	n. d.	↑	↓	= /(↑)	=	(1)
**Lin**	2009	105	SLE vs. HC	n. d.	↑	↑	↑	=	(2)
**Masaki**	2010	95	IgG4-RD vs. SS	=	=	↑	↓	↑↑	(3)
**Zhang**	2015	102	SS, SLE, PBC vs. HC	↑	↑	↓	=	= /(↓)	(4)
**Ueki**	2015	1*	mpGN	n. d.	n. d.	↑*	↑*	Ø	(5)
**Dunkley**	2018	1	IgG4-RD (granulomatosis)	n. d.	n. d.	↑**	n. d.	n. d.	(6)
**Chan**	2017	69	IgG4-ROD vs. non-IgG4-RD	n. d.	n. d.	↑**	n. d.	=	(7)
**Hara**	2016	1	tiGN (IgG4-RD)	n. d.	↑*	↑*	↑*	Ø	(8)
**Yamaguchi**	2012	16	IgG4-RD tiN	*	*	( )*	*	*	(9)

IgG4-RD=immunoglobulin G4 related diseases; SS=Sjögren syndrome; SLE=systemic lupus erythematosus; HC=health controls; PBC=primary biliary cirrhosis, mpGN=membranoproliferative glomerulonephritis; IgG4-ROD=IgG4 related orbital disease, tiN=tubulointerstitial nephritis

*: in tissue; ** serum and tissue
